# Netarsudil as an Adjunctive Therapy: Efficacy and Factors Contributing to a Favorable IOP-Lowering Effect

**DOI:** 10.1155/2022/6925027

**Published:** 2022-12-30

**Authors:** Sandy Samuel, Hani El Helwe, Cameron E. Neeson, Nathan Hall, Ryan Sameen Meshkin, Nino Odishelidze, Rebecca Ye, Ta C. Chang, David Solá-Del Valle

**Affiliations:** ^1^Massachusetts Eye and Ear, Harvard Medical School, Boston, Massachusetts, USA; ^2^Massachusetts Eye and Ear, Boston, Massachusetts, USA; ^3^Bascom Palmer Eye Institute, University of Miami Miller School of Medicine, Miami, FL, USA

## Abstract

**Purpose:**

The aim of the study is to assess netarsudil's intraocular pressure (IOP)-lowering potential when prescribed as an adjunctive agent, to examine the effect of baseline IOP on patients' response to netarsudil, and to explore patients' characteristics predictive of pronounced responses to netarsudil.

**Methods:**

This is a single-center, multiprovider retrospective cohort study set at Massachusetts Eye and Ear. Patients with a diagnosis of glaucoma or ocular hypertension on netarsudil and at least one other hypotensive agent for glaucoma who had at least one month of follow-up were included. Patients with additional procedures or glaucoma medication changes were excluded. The main outcome measures were IOP reduction, Kaplan–Meier survival analyses, netarsudil responder type, and complication rates.

**Results:**

236 eyes of 236 patients were included. The mean baseline IOP was 19.06 ± 4.6 mmHg on an average of 4 ocular hypotensive medications. 196 (83.1%) patients experienced IOP reduction at the first follow-up visit of 2.84 ± 0.30 mmHg at 55.66 ± 51.89 days. IOP reduction at the second visit among these patients was 3.01 ± 0.44 mmHg at 133.24 ± 77.63 days. After starting netarsudil, 59% had a sustained response (median duration of 315 days), 25% had a robust response (>20% IOP reduction for at least 80% of visits), and 10% had a super response (>20% and >10 mmHg IOP reduction). Netarsudil was effective as an adjunctive therapy across all baseline IOP categories with greater relative IOP reduction in higher baseline IOP groups.

**Conclusions:**

Netarsudil is an effective adjunctive glaucoma therapy. IOP reductions between 2 and 3 mmHg are typical, but a minority had more pronounced and sustained effects (>10 mmHg). Further analysis is needed to assess specific demographic and clinical factors predictive of these robust responses.

## 1. Introduction

Netarsudil ophthalmic solution 0.02% [(Rhopressa (Aerie Pharmaceuticals Inc, Durham, NC)], a Rho-associated protein kinase and norepinephrine transporter inhibitor, is a novel class of topical ocular hypotensive medical therapy introduced in 2017. It lowers intraocular pressure (IOP) by simultaneously decreasing trabecular meshwork resistance, aqueous humor formation, and episcleral venous pressure [1], which makes netarsudil a good adjunctive IOP-lowering agent when used with other single-mechanism medications.

When used as monotherapy, netarsudil's average IOP-lowering effect was 5.2–6.6 mmHg [2]. Likewise, pooled analyses of the Rho Kinase Elevated IOP Treatment Trials 1, 2, and 4 (ROCKET-1, -2, and -4) demonstrated noninferiority of netarsudil compared to timolol 0.5% through 3 months among patients with maximum baseline IOP <25 mmHg [2, 3].

The efficacy of netarsudil as adjunctive therapy, however, has only been recently explored in the literature. The MERCURY trials demonstrated that the fixed-dose combination (FDC) of netarsudil (0.01% and 0.02%) and latanoprost (0.005%) lowered IOP more effectively than its individual components [4, 5]. Similarly, a few recent studies have demonstrated netarsudil's efficacy as adjunctive therapy after a treatment duration of 1–6 months [6–8]. For example, Prager et al. noted a modest IOP reduction of 2.2 mmHg (*p* < 0.001) in patients on two or more ocular hypotensive medications, including netarsudil with 16.2% of eyes showing a sustained ≥20% IOP reduction during the first two follow-up visits. However, the magnitude of response to netarsudil addition remains unexplored. If known, these data may help providers anticipate the degree of IOP drop according to the patient's demographic and clinical data.

In this study, we characterized netarsudil's IOP-lowering effect when used as a second, third, fourth, fifth, and sixth agent and characterized the response duration in a survival analysis. We also uniquely identified patient cohorts with a pronounced and sustained response to netarsudil, whom we designated as “Robust long-term responders” (>20% IOP reduction for at least 80% of visits) and “Super responders” (>20% and >10 mmHg IOP reduction). Such a significant IOP reduction has not been extensively reported to our knowledge in any clinical trials or case reports. Finally, we explored the characteristics associated with a favorable IOP-lowering response as well as reasons for netarsudil's discontinuation.

## 2. Methods

### 2.1. Study Design

This is a retrospective cohort study of patients with a diagnosis of glaucoma or ocular hypertension on netarsudil and at least one other hypotensive agent for glaucoma at Massachusetts Eye and Ear (MEE). Approval was obtained from the Mass General Brigham Institutional Review Board, and data collection abided by the Declaration of Helsinki and the Health and Portability and Accountability Act. Medical records of patients who were prescribed netarsudil by a provider at MEE between April, 2018, and August, 2020, were identified and reviewed. Records were included for analysis if they met the following criteria: (1) the presence of at least 2 IOP recordings prior to netarsudil initiation and (2) at least 1 month of follow-up without additional procedures or glaucoma medication changes. For patients treated with netarsudil in both eyes, the right eye was included in our study. Patients were included in the analysis until they experienced one of the following censoring events: netarsudil discontinuation, a change in the number of adjunctive hypotensive medications, intraocular surgery, or laser procedure. Self-reported medication adherence was recorded.

### 2.2. Outcome Measures

The primary outcome measures were intraocular pressure (IOP) reduction (mmHg) from baseline and percent IOP reduction from baseline. Baseline IOP was calculated by averaging the 2 most recent IOP measurements prior to starting netarsudil. IOP measurements were performed with Goldmann applanation tonometry. IOP reduction following netarsudil initiation was assessed at various time intervals which were selected based on a statistical function that evenly distributes office visits across the five chosen time intervals: 1 to 39 days, 40 to 74 days, 75 to 124 days, 125 to 299 days, and 300+ days.

### 2.3. Response Classification

**Figure 1:**
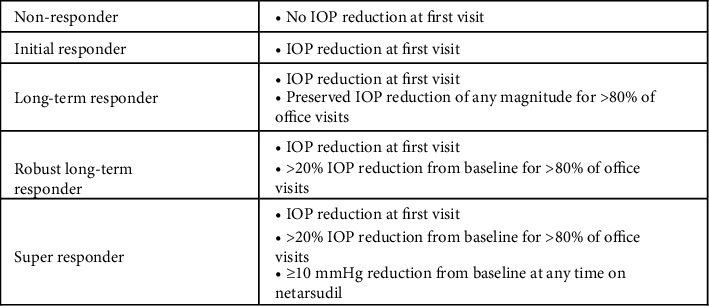
Flowchart defining the different classifications of netarsudil responses.

**Figure 2:**
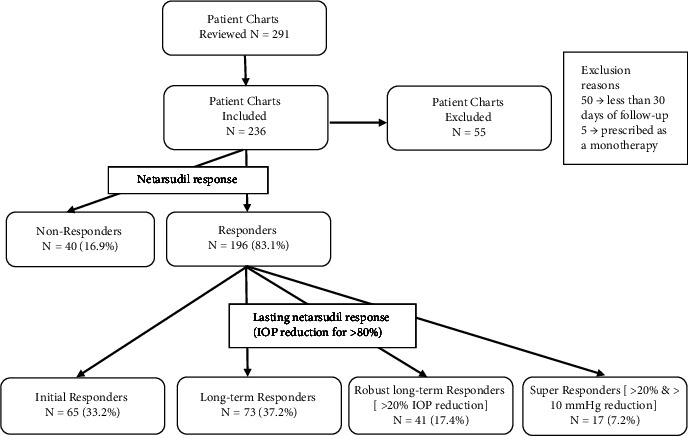
Flowchart of subject inclusion and exclusion in this study and categorization of subjects depending on their response to netarsudil.

The patients' response to netarsudil was defined according to the following designations (Figures 1 and 2). “Nonresponders” were patients who did not experience IOP reduction at the first visit after netarsudil initiation. “Initial responders” were those with IOP reduction at the first follow-up visit after netarsudil initiation who did not maintain IOP reduction from baseline for at least 80% of subsequent visits. We further divided responders who demonstrated sustained IOP response into three groups, which we termed “Long-term responders,” “Robust long-term responders,” and “Super responders,” to highlight netarsudil's pronounced IOP-lowering effect in the latter groups. “Long-term responders” were patients with IOP reduction from baseline at the first visit after netarsudil initiation and for at least 80% of subsequent visits. Patients characterized as “Robust long-term responders” were those with >20% IOP reduction from baseline after netarsudil initiation for at least 80% of visits. Lastly, “Super responders” were those with >20% IOP reduction from baseline after netarsudil initiation for at least 80% of visits who also experienced 10 mmHg or greater IOP reduction at any point while on netarsudil. IOP reduction of 10 mmHg was chosen based on its being twice the standard deviation of IOP reduction seen in the original clinical trials [2]. Given that our responder classifications are time-dependent, the average number of follow-up visits for the total number of subjects and each responder type was as follows: 2.47 ± 2.42 overall, 1.95 ± 2.32 for nonresponders, 2.31 ± 2.44 for initial responders, 2.60 ± 2.39 for long-term responders, 2.59 ± 2.37 for robust long-term responders, and 2.92 ± 2.60 for super responders.

In addition, patients were further divided into different groups based on baseline IOP relative to the mean IOP, which was 19.06 mmHg. “Low” IOP was defined as IOP < 15, “Medium” IOP was 15 ≤ IOP < 20, “High” IOP was 20 ≤ IOP < 25, and “Very high” was defined as IOP ≥ 25 (Table 1).

### 2.4. Statistical Analysis

Primary analyses were unpaired *t*-tests on IOP reduction from baseline at specific time intervals or office visits. Secondary analyses included Kaplan–Meier survival analysis for failure to preserve >20% IOP reduction from baseline for two consecutive visits. Tertiary analyses were logistic regression model outcomes for the odds of a subject being classified as a given responder type. Logistic regression models were fitted using baseline data to model the log odds of subjects belonging to various responder groups of interest. Stepwise selection in both directions utilizing Akaike's information criterion was performed on each fully saturated model to determine a final, “best” model for each outcome. Statistical analysis was performed in R software.

## 3. Results

Overall, 236 eyes of 236 patients were included for analysis, with a mean age of 71.6 ± 14.2 years (range: 10–98 years) with 0.85% of patients falling in the 10–18 years age range, 22.88% in the 19–64 years, and 76.27% in the 65–98 years; 53% were female, and 70% were Caucasian (Tables 1 and 2). Netarsudil was added to a glaucoma regimen as a second (8.5%, 20/236), third (17.4%, 41/236), fourth (33.1%, 78/236), fifth (32.6%, 77/236)), and sixth or later (8.5%, 20/236) medication (Table 2). The medication classes for adjunctive glaucoma agents for the overall population are shown in Table 3. The proportion of patients falling in each responder group were nonresponders (16.9%, 40/236), initial responders (27.5%, 65/236), long-term responders (30.9%, 73/236), robust-long-term responders (17.4%, 41/236), and super responders (7.2%, 17/236) (Table 1). Most patients had severe stage (55.9%, 132/236) primary open-angle glaucoma (63.6%, 150/236) (Tables 1 and 2). On average, netarsudil was prescribed as a part of a regimen of 4 agents. Baseline characteristics were similar across all 5 agent groups, with no statistical significance between-group differences in characteristics other than the glaucoma stage and having prior surgery (Table 2). When comparing responder groups, baseline characteristics were similar as well, except for the glaucoma stage (Table 1). The baseline differences in the glaucoma stage are expected from a statistical standpoint given that we are comparing 4 different glaucoma stage categories across 5 different responder groups with a small sample size in each cross-tabulation. Additionally, super responders were the only responder group with a significantly greater baseline IOP of 26.8 ± 7.1 mmHg (*p* < 0.001), resulting in a significantly greater representation in the “Very High” IOP baseline category, 9 subjects (53%, 9/17), compared to the other responder groups (*p* < 0.001) (Table 1).

### 3.1. Mean IOP Reduction for the Overall Population and the Different Responder Groups

For the first follow-up office visit, the mean duration of netarsudil treatment for all patients was 55.66 ± 52.00 days (1.83 ± 1.71 months). The IOP reduction at the first visit among all patients was 2.84 ± 0.30 mmHg, 14.34% ± 22.19% (*p* < 0.0001) (Table 4). When analyzed by the responder group, all groups that responded to netarsudil demonstrated significant reduction at the first follow-up visit (*p* < 0.0001). Interestingly, nonresponders demonstrated a significant change as well after this visit, but with IOP increasing by an average of 22.44% ± 17.13% (*p* < 0.0001) (Table 4).

For the second follow-up office visit, the mean duration of netarsudil treatment for all patients was 133.24 ± 77.63 days (4.43 ± 2.59 months). IOP reduction at the second visit among all patients was 3.01 ± 0.44 mmHg, 14.94% ± 28.04% (*p* < 0.0001). At this visit, long-term, robust long-term, and super responders all had a significant IOP reduction with long-term and robust long-term responders having an average reduction of 3.53 and 5.66 mmHg, respectively, and super responders having an average reduction of 12.30 mmHg (*p* < 0.0001 for all groups) (Table 4).

**Figure 3:**
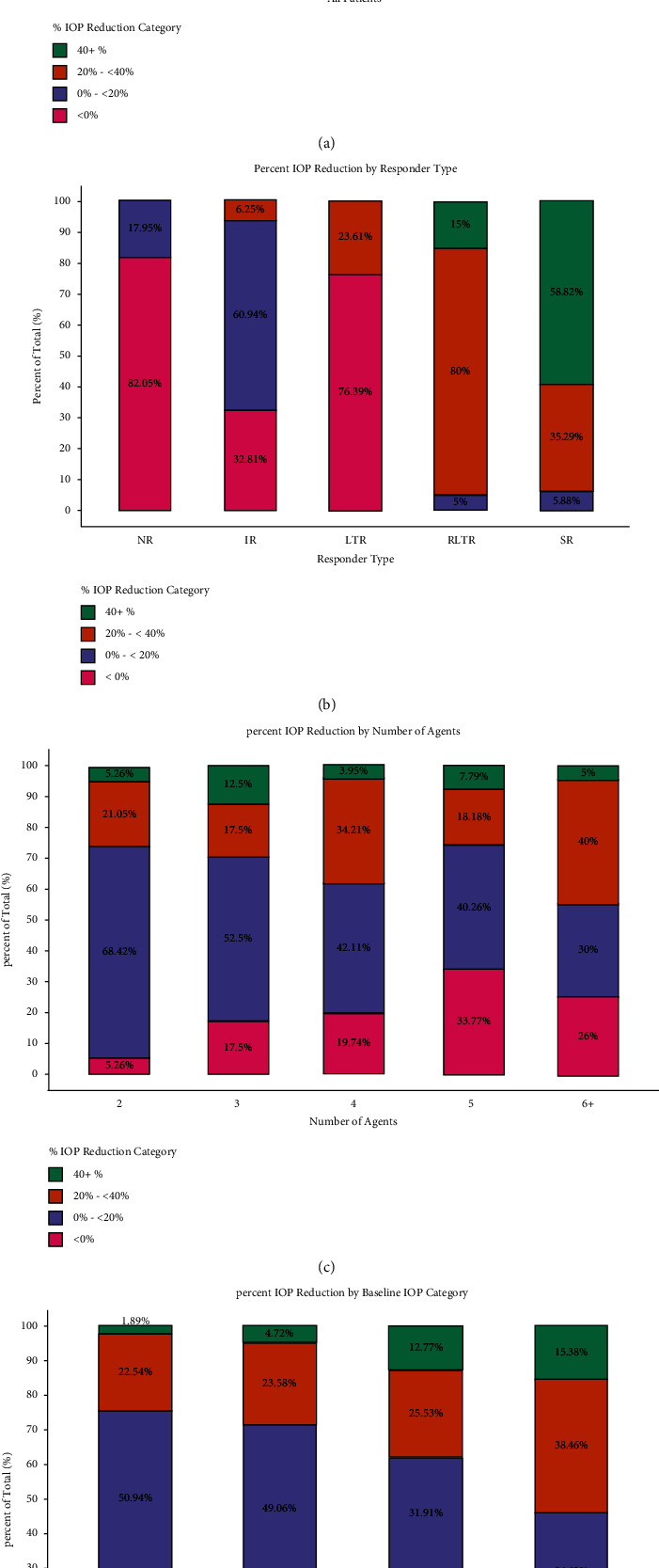
Stacked bar charts for percent IOP reduction from baseline across all patients, number of agents, baseline IOP category, and by the responder type. (a) Percent IOP reduction across all patients. (b) Percent IOP reduction by the number of agents. (c) Percent IOP reduction by the baseline IOP category. (d) Percent IOP reduction by the responder type.

A Tukey test was also conducted to determine if the mean IOP reduction from baseline significantly differed between each responder type. Each of the pairwise Tukey comparisons was significant (*p* < 0.0001 for each comparison), and the overall ANOVA was significant (*p* < 0.0001). Figures 3(a) and 3(b) show the stacked bar chart comparing the IOP reduction from baseline in the overall population and across the different responder types, respectively.

### 3.2. Kaplan–Meier Survival Analysis

**Figure 4:**
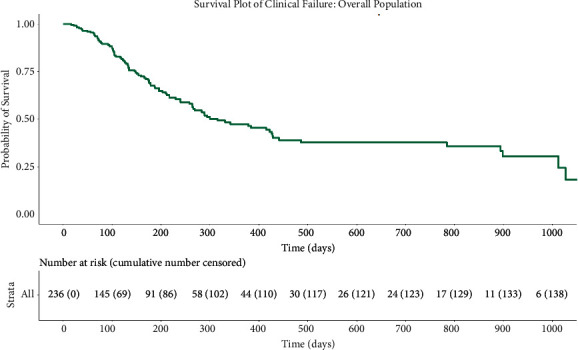
Kaplan–Meier analysis demonstrating a failure to preserve >20% IOP reduction from baseline for two consecutive visits over time in days.

Kaplan–Meier survival analysis for failure to preserve >20% IOP reduction from baseline for two consecutive visits for the overall population is reported in Figure 4. The median survival was 315 days (95% CI 264–429). Patients were censored after they had a censoring event (intraocular surgery or laser procedure, change in regimen, netarsudil discontinuation, or reaching the end of the study period).

Notably, patients who met the super responder criteria (17/236 (7.2%)) maintained >10 mmHg reduction for an average of 218.82 ± 285.59 days (7.19 ± 9.39 months). Failure to preserve the super responder status occurred when patients had an IOP reduction from a baseline of less than 10 mmHg at two consecutive office visits.

A Cox proportional hazards (PH) model analysis was conducted to investigate the effect of age under the same Kaplan–Meier survival criteria. The Cox PH coefficient for age is 0.007 (*p* = 0.39), indicating that age was neither protective nor harmful to survival. We also stratified age into three groups 10–18 years, 19–64, and 65–98 and recalculated the Cox PH coefficient for age as a noncontinuous variable and got a similar result (Supplementary Table 1).

### 3.3. IOP Reduction by Agent Number

A Tukey test was conducted to determine whether there was an association between mean IOP reduction and baseline number of glaucoma medications. None of the pairwise Tukey comparisons were found to be significant. The magnitude of IOP lowering, therefore, did not depend on the number of baseline medications (*p* ≥ 0.771 for all mean comparisons), and the overall ANOVA was not significant (*p* = 0.785). A stacked bar chart was generated to visually compare differences in IOP reduction for the number of glaucoma agents (Figure 3(c)).

### 3.4. IOP Reduction by Baseline IOP

Likewise, a Tukey test was conducted to determine whether the mean IOP reduction depended on the patient's baseline IOP. When comparing means, the “Low,” “Medium,” and “High” baseline IOP categories were each significantly different from the “Very High” baseline IOP category. The mean difference in reduction is −5.29 mmHg when comparing “Low” to “Very High,” −4.42 mmHg for “Medium” to “Very High,” and −3.38 mmHg for “High to “Very High” (*p* < 0.0001, *p* < 0.0001, and *p* = 0.008, respectively); this indicates that those subjects with a “Very High” baseline IOP had a significantly greater mean IOP reduction than any of the other baseline IOP groups. The overall ANOVA was significant (*p* < 0.0001).  Figure 3(d) shows the stacked bar chart comparing the IOP reduction from baseline across the different baseline IOP categories.

### 3.5. Logistic Regression Analysis

Secondary analyses for the odds of a patient being classified as a responder yielded a final, parsimonious model post-stepwise selection using AIC. The findings from five different multiple logistic regression models, representing each responder type, are seen in Table 5. The covariates that were statistically significant were seen across the robust long-term and super responder models. Of note, some odds ratios are presented as reciprocals of those found in Table 5 to allow for easier clinical interpretation. For the super responder model, it was noted that the odds of being a super responder was 76.92 (95% CI: 12.5–100) times that for subjects with a “Very High” baseline IOP categorization compared to “Medium” IOP and 4.02 (95% CI: 0.068–0.827) times that for subjects with a “Very high” baseline IOP categorization as compared to those with a “High” baseline IOP categorization (*p* < 0.001 and *p* = 0.027, respectively). It is important to note, however, the small sample size of patients in this responder group (*n* = 17) with only 1 patient having a “Medium” baseline IOP which reduces the certainty of the true effect size of this association. Finally, the odds of being a super responder was 3.127 (95% CI 0.984–11.078) for patients with a history of prior surgery compared to those without previous eye surgery with our results trending towards significance (*p* = 0.061).

The odds of being a robust long-term responder was 9.353 (95% CI: 1.790–172.813) times that for subjects with a “Medium” baseline IOP categorization as compared to those with a “Very High” baseline IOP categorization, maintaining the other parameters as constant (*p* = 0.034). In addition, the odds of being a robust long-term responder was 2.03 (95% CI: 0.922–4.386) times for patients with a glaucoma type other than POAG compared to those with POAG with our results approaching significance (*p* = 0.074).

### 3.6. Adverse Effects of Netarsudil

The adverse effects experienced across all patients and by responder type are summarized in Table 6. The only adverse effect that demonstrated statistical significance across responder types was tearing (*p* = 0.003), which was the highest in long-term responders.

## 4. Discussion

In our retrospective cohort study, we reported netarsudil's IOP-lowering effect as an adjunctive agent. Our study found an IOP-lowering effect when prescribed as adjunctive therapy with an average reduction from baseline of 2.84 ± 0.30 mmHg (14.34% ± 22.19%) at the first follow-up visit and 3.01 ± 0.44 mmHg (14.94% ± 28.04%) at the second follow-up visit. Our results are consistent with those of previous studies exploring netarsudil's efficacy as a monotherapy and adjuvant therapy. The ROCKET-1 monotherapy clinical trial reported IOP lowering of 3.3–5.0 mmHg (17–22%) at 3 months [2]. When assessing netarsudil's effect in patients on maximally tolerated medical therapy, Villegas et al. noted a mean IOP lowering of −3.53 mmHg (−17%) at the first follow-up visit which occurred at a mean of 4.8 ± 3.5 weeks. Another study analyzing netarsudil's adjunctive effect found a similar IOP reduction of 3.9 ± 4.6 mmHg (17.5 ± 6.0%) in a relatively small sample of 95 eyes [6]. That study, however, was limited by very short follow-up duration (mean duration on netarsudil: 53.7 days) and high variability in follow-up frequency for IOP measurements (data only reported up to three follow-up visits).

Our study included a longer follow-up period compared to the aforementioned studies and thereby offered important insights when assessing the durability of netarsudil's effect. We found that patients experienced a sustained hypotensive effect lasting well beyond one year, with survival analysis demonstrating a 50% probability of preserving >20% IOP reduction at 400 days. Moreover, 59% (144/244) of all patients in the study experienced a sustained response (IOP reduction from baseline at >80% of office visits) to netarsudil. Explanations for the durability of netarsudil's IOP-lowering effect include potential trabecular meshwork remodeling [4, 5, 10–12] and its unique role in blocking profibrotic TGF-beta effects in glaucoma filtration surgery [13–18].

In addition, our study uniquely stratified patients by the change in IOP into different responder types to demonstrate the range of drug effects. We identified a cohort of patients (17/236, 7.2%), referred to in this study as “super responders,” who demonstrated ≥10 mmHg IOP reduction from baseline as well as a sustained ≥20% IOP reduction at >80% of follow-up visits. Such a pronounced IOP reduction has not been previously reported to our knowledge in clinical trials or case reports. Mounting anecdotal accounts from providers with standout cases of pronounced netarsudil hypotensive effect warrant further exploration of baseline characteristics predictive of such a response. Our logistic regression analysis identified that among the characteristics contributing to the super responders' 12.1 mmHg (45.7%) IOP reduction from baseline was having a “Very High” baseline IOP (IOP ≥ 25) and a history of prior eye surgery (*p* = 0.061) (Table 5). The logistic regression analysis further revealed that having a “Medium” baseline IOP and a glaucoma type other than POAG are potential contributors to netarsudil's efficacy in patients who were referred to in this study as “robust long-term responders,” who experienced >20% reduction in IOP in >80% visits. Studies with larger sample sizes should further investigate the effect of the aforementioned variables on netarsudil's efficacy as well as explore mechanisms that potentially mediate these associations.

We also noted that the degree of IOP reduction was not affected by the baseline number of hypotensive agents (Figure 3(c)). This is consistent with a study by Prager et al., which reported no significant differences in IOP reduction from baseline between patients on 1, 2, 3, or 4 glaucoma medications. This finding provides additional support that netarsudil's adjunctive efficacy may stem from its unique mechanisms of action (decreased trabecular meshwork resistance, decreased aqueous humor formation, and decreased episcleral venous pressure) which complement that of other agents.

IOP inclusion criteria for this study were broad (mean baseline IOP: 19.06 ± 4.6). Netarsudil's adjunctive efficacy was apparent across all baseline IOP categories with greater relative IOP reduction in the “High” and “Very High” baseline IOP groups (38.3% and 53.84%) relative to the low and medium groups (24.53% and 28.3%). There are few prior studies that have explored the role of baseline IOP and its effect on netarsudil efficacy. In the FDA clinical trials for netarsudil and netarsudil/latanoprost FDC, Brubaker et al. found a greater proportion of patients with ≥20% IOP reduction in patients with baseline IOP ≥ 25 mmHg when compared to those with baseline IOP < 25 mmHg [19]. Similarly, previous studies report that the absolute hypotensive effects of timolol and latanoprost were related to baseline IOP [20, 21]. Some of these studies, however, noted that netarsudil's IOP-lowering efficacy was unchanged across various baseline pressures [20]. Our findings suggest that as an adjunctive treatment modality, netarsudil's IOP-lowering effect is greater in patients with higher baseline pressures (>25 mmHg).

Similar to prior studies, we found that netarsudil was well tolerated in most patients [2, 22]. In terms of adverse effects, the most common complication studies have found is conjunctival hyperemia with incidence ranging between 30.4% and 60.6% [2, 7]. Our study is consistent with these results as 38.1% of patients were reported to have conjunctival hyperemia (Table 6).

Our study has several limitations, including the absence of a control group. In addition, given that this study was not a clinical trial and instead was aimed at investigating netarsudil's efficacy in a real-world glaucoma practice, there was no wash-out period; therefore, the hypotensive effect cannot be entirely attributed to netarsudil. Furthermore, while the study did control for changes in the glaucoma regimen with its stringent inclusion criteria, it did not account for changes in the glaucoma regimen coinciding with netarsudil's initiation, such as the concurrent discontinuation of other glaucoma medications. Finally, there was no practical way to distinguish normal diurnal fluctuations of IOP from the IOP measured in the clinic as this study focused on the real-world use of netarsudil as an adjunctive medication and was thus subjected to the constraints of regular daily clinic hours which start at 7:00 am and end at 7:00 pm EST.

## 5. Conclusion

Our results support the use of netarsudil as an adjunctive treatment that can provide significant and sustained IOP lowering with once-daily dosing for patients across a broad baseline IOP range and number of baseline medications. Future studies should aim to create more explicit terminology that considers criteria related to netarsudil response over a longer follow-up period as well as further assesses specific demographic and clinical factors predictive of these robust responses.

## 6. Disclosure

The research was conducted as part of the employment of the authors.

## Figures and Tables

**Table 1:** Demographic and ocular data characterized by the responder type.

Variable	Overall *N* = 236^1^	Nonresponder *N* = 40^1^	Initial responder *N* = 65^1^	Long-term responder *N* = 73^1^	Robust long-term responder *N* = 41^1^	Super responder *N* = 17^1^	*p* value^2^
*Demographics*							
Female sex, *N* (%)	125 (53)	20 (50)	35 (54)	40 (55)	24 (59)	6 (35)	0.57
*Age*							0.38
Mean ± SD	71.6 ± 14.2	73.2 ± 13.1	72.6 ± 15.4	69.8 ± 12.8	72.7 ± 15.0	69.0 ± 16.7	
Range	10–98	43–98	10–97	30–91	31–95	31–88	
*Ethnicity, N (%)*							
White	165 (70)	25 (62)	49 (75)	51 (70)	27 (66)	13 (76)	0.36
Black	23 (9.7)	6 (15)	6 (9.2)	9 (12)	2 (4.9)		
Asian	16 (6.8)	3 (7.5)	4 (6.2)	3 (4.1)	3 (7.3)	3 (18)	
Hispanic	7 (3.0)	2 (5.0)		3 (4.1)	1 (2.4)	1 (5.9)	
Other	25 (10.6)	4 (10)	6 (9.2)	7 (9.6)	8 (20)		
*Glaucoma stage, N (%)*							**0.024**
Indeterminate	19 (8.0)		3 (4.6)	10 (14)	2 (4.9)	4 (24)	
Mild	31 (13)	8 (20)	6 (9.2)	10 (14)	5 (12)	2 (12)	
Moderate	55 (23)	8 (20)	18 (28)	21 (29)	5 (12)	2 (12)	
Severe	132 (56)	24 (60)	38 (58)	32 (44)	29 (71)	9 (53)	
*Glaucoma type, N (%)*							0.60
Primary open-angle	150 (64)	28 (70)	42 (65)	48 (66)	24 (59)	8 (47)	
Pseudoexfoliative	28 (12)	5 (12)	9 (14)	8 (11)	3 (7.3)	3 (18)	
Mixed mechanism	20 (8.5)	2 (5.0)	6 (9.2)	5 (6.8)	5 (12)	2 (12)	
Normal tension	11 (4.7)	1 (2.5)	2 (3.1)	4 (5.5)	4 (9.8)		
Chronic angle-closure	10 (4.2)	3 (7.5)	3 (4.6)	1 (1.4)	2 (4.9)	1 (5.9)	
Pigmentary	4 (1.7)	1 (2.5)		2 (2.7)		1 (5.9)	
Uveitic	3 (1.3)			2 (2.7)	1 (2.4)		
Ocular hypertension	3 (1.3)			1 (1.4)	1 (2.4)	1 (5.9)	
Aphakic	2 (0.8)			1 (1.4)		1 (5.9)	
Congenital	2 (0.8)		1 (1.5)	1 (1.4)			
Steroid	2 (0.8)		2 (3.1)				
Neovascular	1 (0.4)				1 (2.4)		
Prior surgery	105 (44)	20	30	28	17	10	0.84
Phaco	89 (85)	18 (90)	24 (80)	25 (89)	13 (76)	9 (90)	
Trabeculectomy	11 (10)	1 (5.0)	5 (17)	2 (7.1)	2 (12)	1 (10)	
Tube shunt	3 (2.9)	1 (5.0)		1 (3.6)	1 (5.9)		
MIGS	2 (1.9)		1 (3.3)		1 (5.9)		
Prior laser	84	16	29	20	12	7	0.16
Laser trabeculoplasty	61 (73)	11 (69)	18 (62)	16 (80)	11 (92)	5 (71)	
CPC/ECP	15 (18)	3 (19)	10 (34)	1 (5.0)		1 (14)	
Iridotomy	8 (9.5)	2 (12)	1 (3.4)	3 (15)	1 (8.3)	1 (14)	
*Number of agents, N (%)*							0.74
2^nd^ agent	20 (8.5)	3 (7.5)	6 (9.2)	7 (9.6)	4 (9.8)		
3^rd^ agent	41 (17)	7 (18)	10 (15)	14 (19)	7 (17)	3 (18)	
4^th^ agent	78 (33)	10 (25)	20 (31)	24 (33)	17 (41)	7 (41)	
5^th^ agent	77 (33)	18 (45)	23 (35)	23 (32)	7 (17)	6 (35)	
6^th^+ agent	20 (8.5)	2 (5.0)	6 (9.2)	5 (6.8)	6 (15)	1 (5.9)	
*Baseline IOP (mmHg)*							
Mean ± SD	18.2 ± 5.2	17.2 ± 3.7	17.3 ± 4.9	18.1 ± 4.8	17.1 ± 3.4	26.8 ± 7.1	**<0.001**
* IOP category, N (%)*							**<0.001**
Low (IOP < 15)	55 (23)	12 (30)	18 (28)	16 (22)	9 (22)		
Medium (15 ≤ IOP < 20)	108 (46)	17 (42)	31 (48)	33 (45)	26 (63)	1 (5.9)	
High (20 ≤ IOP < 25)	47 (20)	10 (25)	10 (15)	15 (21)	5 (12)	7 (41)	
Very high (IOP ≥ 25)	26 (11)	1 (2.5)	6 (9.2)	9 (12)	1 (2.4)	9 (53)	

SD = standard deviation; IOP = intraocular pressure; phaco = cataract surgery; MIGS = microinvasive glaucoma surgery; CPC/ECP = diode cyclophotocoagulation/endoscopic cyclophotocoagulation. ^1^mean (SD); (min-max); median for numerical variables; (% missing), or frequency (%); (% missing) for categorical variables. ^2^Kruskal–Wallis rank-sum test. Pearson's Chi-squared test.

**Table 2:** Demographic and ocular data characterized by the number of glaucoma agents.

Variable	Overall *N* = 236^1^	2^nd^ agent *N* = 20^1^	3^rd^ agent *N* = 41^1^	4^th^ agent *N* = 78^1^	5^th^ agent *N* = 77^1^	6^th^+ agent *N* = 20^1^	*p* value^2^
*Demographics*							
Female sex, *N* (%)	125 (53)	15 (75)	25 (61)	40 (51)	35 (45)	10 (50)	0.14
* Age*							
Mean ± SD	71.6 ± 14.2	72.2 ± 14.6	74.9 ± 12.6	71.3 ± 14.8	70.9 ± 13.8	68.3 ± 16.4	0.34
Range	10–98	30–94	34–94	10–97	13–97	31–98	
* Ethnicity, N (%)*							0.60
White	165 (70)	13 (65)	27 (66)	58 (74)	53 (69)	14 (70)	
Black	23 (9.7)	2 (10)	1 (2.4)	8 (10)	11 (14)	1 (5.0)	
Asian	16 (6.8)		7 (17)	4 (5.1)	4 (5.2)	1 (5.0)	
Hispanic	7 (3.0)	1 (5.0)	1 (2.4)	2 (2.6)	2 (2.6)	1 (5.0)	
Other	25 (10.6)	4 (20)	5 (11.9)	6 (7.6)	7 (9.1)	3 (15)	
*Glaucoma stage, N (%)*							**0.033**
Indeterminate	19 (8.0)	3 (15)	3 (7.3)	7 (9.0)	6 (7.8)		
Mild	31 (13)	7 (35)	3 (7.3)	8 (10)	8 (10)	5 (25)	
Moderate	54 (23)	5 (25)	14 (34)	16 (21)	14 (18)	5 (25)	
Severe	132 (56)	5 (25)	21 (51)	47 (60)	49 (64)	10 (50)	
*Glaucoma type, N (%)*							0.38
Primary open-angle	150 (64)	15 (75)	28 (68)	49 (63)	47 (61)	11 (55)	
Pseudoexfoliative	28 (12)	2 (10)	3 (7.3)	10 (13)	11 (14)	2 (10)	
Mixed mechanism	20 (8.5)	1 (5)	3 (7.3)	7 (9)	6 (7.8)	3 (15)	
Normal tension	11 (4.7)	1 (5)	3 (7.3)	6 (7.7)	1 (1.3)		
Chronic angle-closure	10 (4.2)			3 (3.8)	6 (7.8)	1 (5)	
Pigmentary	4 (1.7)		2 (4.9)		1 (1.3)	1 (5.0)	
Uveitic	3 (1.3)	1 (5)			1 (1.3)	1 (5.0)	
Ocular hypertension	3 (1.3)		2 (4.9)		1 (1.3)		
Aphakic	2 (0.8)				2 (2.6)		
Congenital	2 (0.8)			2 (2.6)			
Steroid	2 (0.8)				1 (1.3)	1 (5.0)	
Neovascular	1 (0.4)			1 (1.3)			
Prior surgery	105 (44)	6	22	32	37	8	**0.021**
Phaco	89 (85)	3 (50)	17 (77)	28 (88)	36 (97)	5 (62)	
Trabeculectomy	11 (10)	2 (33)	4 (18)	3 (9.4)	1 (2.7)	1 (12)	
Tube shunt	3 (2.9)	1 (17)		1 (3.1)		1 (12)	
MIGS	2 (1.9)		1 (4.5)			1 (12)	
Prior laser	84	4	15	25	31	9	0.51
Laser trabeculoplasty	61 (73)	4 (100)	12 (80)	18 (72)	19 (61)	8 (89)	
CPC/ECP	15 (18)		1 (6.7)	4 (16)	9 (29)	1 (11)	
Iridotomy	8 (9.5)		2 (13)	3 (12)	3 (9.7)	0 (0)	
*Responder type, N (%)*							0.74
Nonresponder	40 (17)	3 (15)	7 (17)	10 (13)	18 (23)	2 (10)	
Initial responder	65 (28)	6 (30)	10 (24)	20 (26)	23 (30)	6 (30)	
Long-term responder	73 (31)	7 (35)	14 (34)	24 (31)	23 (30)	5 (25)	
Robust long-term responder	41 (17)	4 (20)	7 (17)	17 (22)	7 (9.1)	6 (30)	
Super responder	17 (7.2)		3 (7.3)	7 (9.0)	6 (7.8)	1 (5.0)	
*Baseline IOP (mmHg)*							
Mean ± SD	18.2 ± 5.2	17.5 ± 5.6	17.2 ± 4.1	17.9 ± 5.3	18.7 ± 5.5	20.1 ± 4.8	0.088
* IOP category, N (%)*							0.63
Low (IOP < 15)	55 (23)	6 (30)	9 (22)	22 (28)	17 (22)	1 (5.0)	
Medium (15 ≤ IOP < 20)	108 (46)	9 (45)	22 (54)	32 (41)	35 (45)	10 (50)	
High (20 ≤ IOP < 25)	47 (20)	3 (15)	7 (17)	18 (23)	14 (18)	5 (25)	
Very high (IOP ≥ 25)	26 (11)	2 (10)	3 (7.3)	6 (7.7)	11 (14)	4 (20)	

SD = standard deviation; IOP = intraocular pressure; phaco = cataract surgery; MIGS = microinvasive glaucoma surgery; CPC/ECP = diode cyclophotocoagulation/endoscopic cyclophotocoagulation. ^1^mean (SD); (min-max); median for numerical variables; (% missing), or frequency (%); (% missing) for categorical variables. ^2^Kruskal–Wallis rank-sum test. Pearson's Chi-squared test.

**Table 3:** Medication class representation for the adjunctive glaucoma agent(s) for total population.

Medication Classes	*N * ^1^ (%^2^)
Prostaglandin analog	183 (77.54)
Alpha_2_ agonist	126 (53.39)
Beta-adrenergic antagonist	197 (83.47)
Carbonic anhydrase inhibitor	178 (75.42)
Cholinergic agonist	36 (15.25)

^1^Percentage of subjects out of the total population who took medication that fell in the respective medication class. ^2^Number of subjects that took a medication in the relevant medication class.

**Table 4:** IOP reduction at first and second visits.

	Total responders	Nonresponders	Initial responders	Long-term responders	Robust long-termresponders	Super responders
First visit (mmHg)	−2.84 ± 0.30	3.80 ± 0.50	−2.79 ± 0.37	−3.07 ± 0.27	−5.16 ± 0.32	−11.41 ± 0.98
First visit (%)	−14.18 ± 22.16	22.40 ± 17.13	−14.71 ± 12.37	−16.85 ± 10.97	−29.53 ± 9.01	−45.56 ± 15.59
First visit *p* value	**<0.0001**	**<0.0001**	**<0.0001**	**<0.0001**	**<0.0001**	**<0.0001**
Second visit (mmHg)	−3.01 ± 0.44	0.63 ± 1.08	0.05 ± 0.87	−3.53 ± 0.39	−5.66 ± 0.33	−12.30 ± 1.10
Second visit (%)	−14.94 ± 28.04	4.13 ± 27.99	1.02 ± 32.18	−18.71 ± 11.76	−34.27 ± 9.78	−48.13 ± 16.89
Second visit *p* value	**<0.0001**	0.5637	0.9583	**<0.0001**	**<0.0001**	**<0.0001**

mmHg = millimeters of mercury. Mean and SD Rhopressa duration (in days) at instance 1: 55.66 ± 51.89 days. Mean and SD Rhopressa duration (in days) at instance 2: 133.24 ± 77.63 days.

**Table 5:** Logistic regression analysis exploring pertinent covariates of responder status through stepwise selection using AIC criteria^1^.

Model	*Model 1: initial responder*			*Model 2: long-term responder*			*Model 3: robust long-term responder*			*Model 4: super responder*		
	Odds ratio	95% CI	*p*value	Odds ratio	95% CI	*p* value	Odds ratio	95% CI	*p* value	Odds ratio	95% CI	*p* value
Intercept	0.31	0.211–0.446	<0.001	0.535	0.381–0.744	<0.001	0.061	0.003–0.303	0.007	0.36	0.130.0.882	0.033
Glaucoma type: POAG (vs. other)							0.493	0.228–1.085	0.074			
Glaucoma type: PFX (vs. other)							0.32	0.067–1.143	0.105			
Had prior laser	1.699	0.944–3.051	0.076	0.584	0.314–1.055	0.08						
Had prior surgery										3.127	0.984–11.078	0.061
Low baseline IOP (vs. very high)							5.573	0.938–107.04	0.116	0	N/A	0.989
Medium baseline IOP (vs. very high)							9.353	1.790–172.81	0.034	0.013	0.001–0.080	<0.001
High baseline IOP (vs. very high)							3.221	0.476–63.997	0.3	0.249	0.068–0.827	0.027

CI = confidence interval; IOP = intraocular pressure; POAG = primary open-angle glaucoma; PXG = pseudoexfoliative glaucoma. ^1^Stepwise-selected “best” models created from the full model, which include all of the possible baseline covariates (seen in Table 1). The automated procedure chooses the best model, which is the best combination of the aforementioned covariates. ^2^CI cannot be estimated here since 0 super responders fall into the “low” baseline IOP category. ^3^IOP baseline categories: low (IOP < 15 mmHg), medium (15 mmHg ≤ IOP < 20 mmHg), high (20 mmHg ≤ IOP < 25 mmHg), and very high (IOP ≥ 25 mmHg).

**Table 6:** Adverse effects.

Variable, *N* (%)	Overall *N* = 236^1^	Nonresponder *N* = 40^1^	Initial responder *N* = 65^1^	Long-term responder *N* = 73^1^	Robust long-term responder *N* = 41^1^	Super responder *N* = 17^1^	*p* value^2^
Problem with adherence	22 (9.3)	6 (15)	6 (9.2)	4 (5.5)	5 (12)	1 (5.9)	0.49
Conjunctival hyperemia	90 (38)	13 (32)	26 (40)	27 (37)	16 (39)	8 (47)	0.87
Blurred vision	50 (21)	5 (12)	17 (26)	17 (23)	8 (20)	3 (18)	0.53
Tearing	40 (17)	3 (7.5)	6 (9.2)	22 (30)	8 (20)	1 (5.9)	**0.003**
Itching	19 (8.1)	2 (5.0)	5 (7.7)	8 (11)	3 (7.3)	1 (5.9)	0.83
Pain	20 (8.5)	2 (5.0)	3 (4.6)	6 (8.2)	5 (12)	4 (24)	0.11
Irritation	30 (13)	4 (10)	5 (7.7)	12 (16)	8 (20)	1 (5.9)	0.28
Burning	9 (3.8)	1 (2.5)	2 (3.1)	3 (4.1)	3 (7.3)		0.67
Dry eye	28 (12)	5 (12)	4 (6.2)	8 (11)	9 (22)	2 (12)	0.19
Headache	11 (4.7)	3 (7.5)	2 (3.1)	5 (6.8)	1 (2.4)		0.53
Eyelid redness	15 (6.4)	3 (7.5)	2 (3.1)	6 (8.2)	3 (7.3)	1 (5.9)	0.78
Eyelid edema	12 (5.1)	4 (10)	1 (1.5)	5 (6.8)	2 (4.9)		0.28

^1^Mean (SD); (min-max); median for numerical variables; (% missing), or frequency (%); (% missing) for categorical variables. Subjects may have more than one adverse event; therefore, the number of observations is greater than the number of reported subjects. ^2^5-sample test for equality of proportions without continuity correction.

## Data Availability

The data used to support the findings of this study are available upon request; please contact sandy.samuel96@gmail.com for access.

## Supplementary Materials

Supplementary Materials Supplementary Table 1: Cox proportional hazards model analysis for age showing that age was neither protective nor harmful to survival.
