# Supplementary material for: Netarsudil as an Adjunctive Therapy: Efficacy and Factors Contributing to a Favorable IOP-Lowering Effect
Source: J Ophthalmol. 2022 Dec 30;2022:6925027. doi: 10.1155/2022/6925027 (PMC9822751; doi:10.1155/2022/6925027)
Supplement: Supplementary Materials — Supplementary Table 1: Cox proportional hazards model analysis for age showing that age was neither protective nor harmful to survival. [file 6925027.f1.docx]

| **Variable type** | **Age range** | **Cox PH Coefficient** | **p-value** | **Number of patients** | **Percentage (%)** |
| --- | --- | --- | --- | --- | --- |
| *Continuous* | **10-98** | 0.007 | 0.39 | 236 | 100.00 |
| *Stratified* | **10-18** | n/a | n/a | 2 | 0.85 |
| *(non-* | **19-64** | 0.055 | 0.96 | 54 | 22.88 |
| *continuous)* | **65-98** | 0.282 | 0.79 | 180 | 76.27 |

**Supplementary Table 1: Cox proportional hazards (PH) model analysis for age**

PH = proportional hazards; n/a = not available
